# Status of vaccine research and development for *Helicobacter pylori*

**DOI:** 10.1016/j.vaccine.2018.01.001

**Published:** 2019-11-28

**Authors:** Philip Sutton, Joanne M. Boag

**Affiliations:** a Murdoch Children’s Research Institute, Royal Children’s Hospital, Parkville, Victoria 3052, Australia; b Centre for Animal Biotechnology, Faculty of Veterinary and Agricultural Science, University of Melbourne, Parkville, Victoria 3010, Australia; c Department of Paediatrics, University of Melbourne, Parkville, Victoria 3010, Australia

**Keywords:** *Helicobacter pylori*, Vaccine, Research and development

## Abstract

Gastric adenocarcinoma is globally the third leading cause of death due to malignancy, with the bulk of this disease burden being suffered by low and middle income countries (LMIC), especially in Asia. The majority of these cancers develop as a result of a chronic gastritis that arises in response to infection with the stomach-dwelling bacterium, *Helicobacter pylori*. A vaccine against this pathogen would therefore be a powerful tool for preventing gastric adenocarcinoma. However, notwithstanding a proof-of-concept that vaccination can protect children from acquisition of *H. pylori* infection, there are currently no advanced vaccine candidates with only a single vaccine in Phase I clinical trial. Further, the development of a vaccine against *H. pylori* is not a current strategic priority of major pharmaceutical companies despite the large global disease burden. Given the involvement of such companies is likely to be critical for late stage development, there is therefore a need for an increased appreciation of the burden of this disease in LMIC and more investment to reinvigorate research in *H. pylori* vaccine Research and Development.

## 1 *Helicobacter pylori* and associated diseases

*Helicobacter pylori* infections of the stomach mucosa typically start in childhood and, unless treated, normally remain for life. The route of *H. pylori* transmission has not been definitively proven and remains controversial. Evidence suggests oral-oral as a likely common route of transmission; this is supported by the ability to cultivate *H. pylori* from vomitus or the oral region and analyses of bacterial strains which have indicated common vertical transmission between mothers (but not fathers) and their children [1], [2]. However transmission by the faecal-oral route is also possible, particularly in areas of poor sanitation (reviewed in [3]).

While an estimated one half of the world’s population is infected with *H. pylori*, the levels of infection are considerably greater in countries of low socio-economic status (a major risk factor for *H. pylori* infection) [4]. Infection levels have slowly declined in most developed nations over the past few decades. This now appears to also be occurring in many developing countries, although there remain high levels of infection in many countries, particularly in Asia. This decline however does not seem to be universal even within a country, indicating regional effects, for example the prevalence of *H. pylori* infection was reported as considerably lower in urban compared to rural China (47% versus 66% respectively) in a recent systematic review [5]. As *H. pylori* infection levels might be stabilizing in developed nations [5], [6] and given the high rates of associated disease in developing countries (see below), it would be dangerous to assume the trend of declining infection rates will continue.

Infection with *H. pylori* is a proven cause of a range of diseases including gastric and duodenal ulcers (peptic ulcer disease), gastric MALT (mucosal associated lymphoid tissue) lymphoma, and gastric adenocarcinoma (hereafter gastric cancer) [7], [8]. There is reasonable evidence that this infection is also a cause of at least some cases of immune thrombocytopenic purpura (ITP) [9], [10]. With the exception of ITP, *H. pylori*-associated diseases are the result of the extremely chronic gastritis that results from the infection.

The most serious consequence of *H. pylori* infection, and the key reason a vaccine is required, is gastric cancer which globally is the 3rd leading cause of death due to cancer [11]. Gastric cancer is the 5th most common malignancy (∼952,000 cases in 2012, 6.8% of all cancers), and the 3rd leading cause of cancer-related deaths worldwide (∼723,000 deaths in 2012, 8.8% of all cancers) [11]. The prevalence of gastric cancer is particularly high in Asian countries, especially China (where 42% of all new cases of gastric cancer have been reported to occur) [12], Japan and South Korea, where *H. pylori* infection has a high prevalence. *H. pylori* is responsible for the majority of non-cardia gastric cancers, the predominant type of cancers in the stomach [13], [14]. There is also considerable morbidity and mortality associated with peptic ulcer disease in many low and middle income countries (LMIC), although as these are also caused by the overuse of non-steroidal anti-inflammatory drugs, it is not always clear how much is due to *H. pylori* infection.

*H. pylori* infections are currently treatable with combination antimicrobial therapies, although antibiotic resistance is a major concern. Antimicrobial therapy has been considered at a population level in China where trials are ongoing; for example in a recent trial of >184,000 people with a prevalence of infection of 57%, an eradication rate of ∼73% was achieved [12]. A failure rate of over one in four, in addition to almost half the volunteers not completing the trial [12], suggest significant challenges for population-wide implementation of antimicrobial eradication of *H. pylori*. Antimicrobial eradication is also not expected to protect against reinfection, although this would likely occur at a relatively low rate in adults.

An effective vaccine against *H. pylori* therefore remains a preferential option, especially for LMIC who would particularly benefit given the high prevalence of disease associated with this infection in these countries and the likely lower costs of a vaccine approach as compared to other strategies. There have been a large number of previous reviews, many recent, that provide summaries of earlier efforts to produce an effective *H. pylori* vaccine, as well as discourse on our understandings of the mechanism of action of previous vaccine attempts (some examples are provided) [15], [16], [17], [18], [19], [20], [21]. In this review therefore, we will focus on current attempts to develop such a vaccine.

The primary target group for such a vaccine would be the populations of countries with a high prevalence of *H. pylori*-associated gastric cancer, which include China, South Korea, Russia and Japan. However an anti-*H. pylori* vaccine would have a number of additional benefits, including protecting against peptic ulcer disease and MALT lymphoma. Given these diseases are thought to be the result of the chronic inflammation caused by infection, it is conceivable that a vaccine would not necessarily need to eradicate the infection, but could be an immunomodulatory vaccine that prevents gastritis.

One critical question is whether the vaccine would be given prophylactically or therapeutically. A key issue in this regard is that while infection predominantly commences from very early in life [22], gastric cancer commonly develops from 50 years of age onwards. Hence a prophylactic vaccine would likely need to be given to children in the first few years of life (to reach the maximum number of the target group while uninfected) but health benefits would occur 5 decades later. In a clinical trial in China that tested a prophylactic vaccine in children aged 6–14, 20% of potential vaccines were excluded as they were already infected with *H. pylori* upon pre-screening.[23] Given an estimated 42% of the Chinese population is infected with *H. pylori* this actually represents potentially half of the individuals that need to be protected. This therefore demonstrates that a prophylactic vaccine against *H. pylori* will need to be delivered to very young children if it is to provide a high level of protection.

An effective therapeutic vaccine however could be given at almost any age and still potentially protect against gastric cancer [24], although it would perhaps ideally be given before the 4th decade of life to maximize the chance of preventing cancer. The choice of prophylactic versus therapeutic may be critically important as *H. pylori* can induce a number of immunosuppressive effects that helps to facilitate chronic colonisation [25], [26], [27]; such features from an established infection would potentially oppose the efficacy of a therapeutic vaccine. For this reason, protection following therapeutic vaccination is generally considered to be more difficult to achieve than prophylactic vaccination. However, proof-of-concept has been shown for reducing colonisation by vaccinating mice already infected with *H. pylori* [28], [29], although there is as yet no evidence of therapeutic efficacy against *H. pylori* in humans.

Importantly, while there are few definitive studies, a vaccine against *H. pylori* that reduces the incidence of gastric cancer will likely be cost-effective in both developed countries and in LMIC. A number of reports have indicated that strategies that screen a population for *H. pylori* then treat are cost-effective, using a threshold of US$50,000 per life-year saved [13]. Additionally, an evaluation of prophylactic vaccination against *H. pylori* was determined to be cost-effective in the USA, a country with a relatively low burden of disease [30]. Given this, these data indicate that vaccinating against *H. pylori* would be expected to be cost-effective in LMIC with the highest burden of disease.

## 2 Overview of current efforts

### 2.1 Biological feasibility for vaccine development

*H. pylori* infect during childhood and are typically present for life, despite a vigorous host immune response which includes the invading pathogen being coated with antibodies [31]. This bacterial longevity indicates the development, on the part of the pathogen, of a range of processes for evading effective host immunity [19].

There is evidence that some children do spontaneously clear *H. pylori* infection, in a process that is affected by socioeconomic status [32]. However as this clearance is at least partially due to incidental antibiotic treatment, it is not necessarily evidence of naturally acquired immunity, but may rather be drug related. The best evidence for natural immune-mediated protection against *H. pylori* infection came from a clinical trial where volunteers were vaccinated then challenged with live bacteria; while the vaccine was ineffective, a proportion of volunteers spontaneously cleared the *H. pylori* challenge via a mechanism that was associated with a T-helper cell response [33].

The development of a vaccine to prevent or eradicate *H. pylori* infection has proven extremely challenging. While vaccinating with a surprisingly wide range of antigens, adjuvants and delivery systems can produce a modest reduction in *H. pylori* colonization levels in mice (reviewed in [20], [34]), an approach to produce reliable sterilizing immunity in animal models has not been developed. Of particular importance was the demonstration in mice of the ability to produce protection following therapeutic vaccination [28]; this was probably the first demonstration of therapeutic vaccine efficacy against any bacterial pathogen.

However attempts to translate this partial success in animal models to clinical trial have, with a single exception, proven unsuccessful. The majority of early *H. pylori* vaccine clinical trials focused on the urease antigen with different adjuvants, routes and delivery systems generally proving ineffective in humans [21]. A different approach taken to Phase I trial by Novartis combined three different virulence factors (cagA, vacA and NAP) injected intramuscularly with alum; while shown to be immunogenic [35], this vaccine was subsequently not pursued.

The exception mentioned above was a Phase III clinical trial performed by Wuhu Kangwei Biological Technology of China [23]. The vaccine tested in that trial also contained urease, but was administered orally as a fusion protein joined to the B subunit of heat labile toxin from *Escherichia coli*. This was the first trial to vaccinate children, to follow vaccinees over a period of years, to study the acquisition of natural infection and to show evidence of protection against *H. pylori* infection. The trial reported that the vaccinations reduced the natural acquisition of *H. pylori* at one-year post-vaccination by 71.8%. Some study participants were followed for an additional two years, when protection was reported to fall to 55%. This trial provided proof of concept that a vaccine can protect against natural *H. pylori* infection. As the results of this trial were published in 2015, there is a perception that this study is recent and part of an ongoing vaccine program. However, it was in fact performed between 2005 and 2007 and the development of this vaccine has been discontinued.

### 2.2 General approaches to vaccine development for this disease for low and middle income country markets

Current vaccine strategies under development are generally aimed at eradicating *H. pylori* in order to prevent gastric cancer. Given the majority of cases of gastric cancer occur in LMIC, the development of such a successful vaccine would be expected to have a major health benefit in these countries. The focus of current and previous studies also seem to generally be aimed at prophylactic vaccination, which would necessitate childhood vaccination, as early in life as possible, in order to protect before acquisition of infection.

Given the early stage of the vaccines which are currently under development, it is not clear which geographical group will be targeted although it would be expected that this would include countries with high levels of gastric cancer such as China, South Korea and Russia. This means that late stage clinical trials will most likely/preferentially be performed in such LMIC as this will allow the testing of vaccine efficacy in the main target population group, as well as potentially be essential in order to provide sufficient individuals who will either become (prophylactic vaccine) or who already are (therapeutic vaccine) infected with *H. pylori*.

## 3 Technical and regulatory assessment

A number of animal models are available for examining the efficacy of putative *H. pylori* vaccines. The mouse *H. pylori* model has some value for initial screening of vaccine candidates, but may have limitations; vaccines shown to have some efficacy in mice have not been effective in clinical trial. This may be due to the vaccines being tested being insufficiently protective in the mouse before progressing to clinical trial, or it may reflect a degree of poor translatability of protective efficacy between the mouse model and humans, although limitations of the human vaccine studies cannot also be excluded. Either way, there are potential benefits to using an intermediate large animal model to confirm mouse observations before progressing to clinical trial. The Rhesus macaque (*Macaca mulatta*) provides a quality non-human primate model; captive colonies of macaques are readily (if not already) infected with *H. pylori* and can be used to screen candidate vaccines [36]. Such studies would provide useful data for applications for clinical/regulatory approval prior to human trials.

A key clinical readout for any conventional *H. pylori* vaccine would be protection against either acquisition (prophylactic) or eradication (therapeutic) of infection. A useful tool which has been developed and proven valuable for early clinical trials is the *H. pylori* challenge strain [37]. This strain, which lacks key pathogenicity factors and that is susceptible to a range of antimicrobials so as to be easily eradicated with antibiotics, has already been used in a Phase I trial to challenge vaccine recipients [33]. Later Phase trials of vaccines aimed at eradicating *H. pylori* would almost certainly need to measure the effects of prophylactic or therapeutic vaccination on naturally acquired infection, as performed by the published Phase III study from China [23].

Another significant challenge to the development of an effective vaccine is that there is no known/accepted correlate of protection: for the induction of sterilising immunity it was originally expected that such protection would be antibody mediated and that may still prove to be the case, but the limited protection generated in mice at least does not appear to involve antibodies [29], [38].

## 4 Current status of *H. pylori* vaccine R&D activities

In recent years, a number of new *H. pylori* vaccine development programs have been initiated, predominately by small companies and academic institutions (summarised in Table 1). To our knowledge, no large pharmaceutical/biotechnology company has a current *H. pylori* vaccine development program. All *H. pylori* vaccines currently in development are very early stage (Phase I or preclinical). These vaccines are predominantly composed of purified or recombinant components of *H. pylori* antigens with an adjuvant. Some of these are discussed briefly below.

**Table 1:** Development status of current vaccine candidates.

Candidate Name/Identifier^a^	Preclinical	Phase I	Phase II	Phase III	Ref.
Wuhu Kangwei Biological technology UreB/LTB fusion vaccine				X Discontinued	[23]
Imevax/IMX101		X			[26]
EpiVax/*H. pylori* vaccine	X				[39]
Helicovaxor®	X				[40]
Sichuan University/Urease epitope vaccine	X				[41]
Southern Medical University/Lp220 vaccine	X				[42]
China Pharmaceutical University/Probiotic vaccine delivery	X				[43]
MCRI/Gastric Cancer Vaccine	X				[44]

a This list is not intended to be exhaustive and does not include all recent studies involving vaccines which have produced a reduction of *H. pylori* colonisation in mice.

Preclinical: EpiVax *Helicobacter pylori* vaccine is an epitope-based vaccine, which involves an initial DNA vaccination followed by a peptide-liposome and which has shown some therapeutic protection in mice [39]. Helicovaxor® has examined two approaches, one involving a non-pathogenic *Vibrio cholerae* vaccine strain, engineered to express *H. pylori* antigens (HpaA, UreB and FlaA) [40], and an inactivated strain of *H. pylori*. Recently, two early preclinical studies of *H. pylori* antigen epitope vaccines from Sichuan University (urease subunits) and Southern Medical University (Lp220) have shown limited protection in BALB/c mice [41], [42]. Another approach tested in recent years has utilised probiotics as a vaccine delivery system. Specifically some protection against *H. pylori* colonisation was shown in mice orally dosed with *Lactococcus lactis* recombinantly expressing cholera toxin B subunit and epitopes of *H. pylori* urease [43], although this did not seem to provide any improvement on the protection typically seen with standard recombinant antigen approaches. Finally, the Murdoch Children’s Research Institute (MCRI) is developing a vaccine that rather than aiming to eradicate *H. pylori*, inhibits the inflammation that causes associated disease [44].

In clinical trial: One potentially important novel approach is that of Imevax, who have completed a Phase I clinical trial with IMX101, a vaccine that comprises the *H. pylori* antigen γ-glutamyltranspeptidase (GGT), an outer membrane protein and a mucosal adjuvant. A key reason previous vaccines have likely failed to produce complete protection are the immune evasion strategies possessed by *H. pylori* [19]. One of the most important of these is GGT which appears to have quite potent immunosuppressive activity [25], [26], [45]. Hence this vaccine aims to target and neutralize this defense mechanism, potentially allowing for the generation of a more effective protective immune response to be raised against the other antigenic components of the vaccine. The important feature of this trial is that it is attempting to overcome one mechanism by which *H. pylori* evades host immunity, which might allow an effective vaccine-mediated response to be developed. This is a novel and interesting approach. Given these bacteria appear to have several such defensive strategies, it remains to be seen whether targeting one such mechanism will be sufficient, but it would seem to be a valid strategy worthy of investigation. While the trial is completed, results are not yet available.

As mentioned above, Wuhu Kangwei Biological Technology (renamed Wuhu Kangwei biological technology Co., Ltd. in 2011) has relatively recently published the results of a phase III clinical trial of a *H. pylori* vaccine which showed efficacy against natural acquisition of infection [23], [46]. This study provided an important proof of concept that vaccine-induced protection against *H. pylori* infection is possible. However, while published in 2015, the trial was actually performed between 2005 and 2007 and development of this vaccine has been discontinued.

## 5 Likelihood for financing

The hepatitis B and C viruses, human papillomavirus and *H. pylori* are the three most common causes of cancer attributed to infection. GAVI currently provides support to eligible countries for the hepatitis B and human papillomavirus vaccines, suggesting the possibility for financing of an *H. pylori* vaccine if cost-effectiveness and public health impact can be demonstrated in the contexts of GAVI eligible countries, many of which are in East and Central Asia with the highest burden of *H. pylori* infection and disease. However, several high burden countries are self-procuring, such as China, Korea and Japan, and these have substantial private as well as public markets. The development of an *H. pylori* vaccine is unlikely to proceed without significant investment from large pharmaceutical companies for late stage development. A more robust evaluation of the global disease burden and development of the value proposition for a prophylactic and/or therapeutic vaccine may help to incentivize this investment, considering the scale of the potential market of a preventative of *H. pylori* associated gastric cancer.

## 6 Closing remarks

In summary, there is a considerable unmet need for a vaccine to protect against diseases associated with *H. pylori* infection, and specifically gastric cancer which is particularly needed in a large number of LMIC.

An interesting observation that has emerged over the last decade has been that *H. pylori* infection might protect against a number of apparently unrelated diseases. While evidence for this is still accumulating, and the level of this effect is also not yet completely certain, a protective role of *H. pylori* infection appears possible for a number of conditions including oesophageal reflux disease and oesophageal cancer (likely mediated by *H. pylori* suppression of gastric acid secretion), inflammatory bowel disease and asthma [47], [48], [49]. Hence if/when an effective vaccine against *H. pylori* is developed and implemented, it will likely be important to monitor for any negative effects that the removal of *H. pylori* protection may have on other conditions.

The development of such a vaccine has encountered significant issues; this is reflected in the current status where there is no advanced program and only one vaccine in active clinical development which has recently completed a Phase I clinical trial. There is a clear and considerable need for major investment in this area. Given the unsuccessful vaccine development program to date, what is most needed are completely innovative approaches to overcome the barriers to the development of this vaccine, in particular those posed by the powerful ability of *H. pylori* to evade the hosts’ immune attack [19].
